# A corpus-based comparison of syntactic complexity in academic writing of L1 and L2 English students across years and disciplines

**DOI:** 10.1371/journal.pone.0292688

**Published:** 2023-10-09

**Authors:** Chen Shen, Jirong Guo, Penghai Shi, Sheming Qu, Jiwei Tian

**Affiliations:** 1 Department of Foreign Languages, Xi’an Jiaotong University City College, Xi’an, Shaanxi, China; 2 School of Foreign Studies, Xi’an Jiaotong University, Xi’an, Shaanxi, China; 3 School of Foreign Languages, Chang’an University, Xi’an, Shaanxi, China; 4 School of English Teacher Education, Xi’an International Studies University, Xi’an, Shaanxi, China; 5 ATC Navigation College, Air Force Engineering University, Xi’an, Shaanxi, China; The University of Auckland, NEW ZEALAND

## Abstract

Syntactic complexity, widely acknowledged as a key predictor of writing quality, has gained increasing attention in the realm of academic writing. A notable line of inquiry has centered on the factors that potentially influence syntactic complexity in academic writing. Instead of attending to one factor, the study focuses on multiple factors to examine how language background affects syntactic complexity across years and disciplines. Specifically, it compares the syntactic complexity in English academic writing between L1 English and L1 Chinese university students across four years and/or three disciplines. Fine-grained indices, which include five clausal indices and eight phrasal indices, are utilized to capture specific syntactic features for a full understanding and description of the syntactic preferences shown by L1 English and L1 Chinese students in academic discourse. The results revealed that L1 English students tended to produce more noun phrases in academic writing than L1 Chinese students with the increase of years. Additionally, both cohorts of students followed similar syntactic patterns in cross-disciplinary contexts, with highly frequent use of complex nominals in the Physical Sciences and clausal structures in the Social Sciences. In instances where language background, year, and discipline interact, texts produced by Chinese English learners exhibited reliance on the syntactic features of adverbial clauses, attributive adjectives, and pre-modifying nouns across all disciplines in the early years of their academic journey. Ultimately, some pedagogical implications for academic writing courses are put forward in an attempt to provide valuable insights for enhancing academic literacy among both L1 and L2 students.

## Introduction

Syntactic complexity, referring to the extent of diversity and sophistication of sentential forms in language output [1, 2], has been under the spotlight in the instruction and research of second language (L2) writing. This is due to the recognition that improving writing performance entails the ability of learners to comprehend a variety of syntactic structures and to use them appropriately in diverse contexts [1]. A sizable body of scholarship in this line has sought to find valid and reliable syntactic complexity indices to measure writing quality [3–7], language development [8–11], and proficiency levels [2, 12–14] for predictive purposes. Large-grained indices that focus on complexity at the sentence and clause level (e.g. mean length of T-unit, dependent clauses per T-unit, and complex nominals per clause) have gained widespread and longstanding use [1, 2, 15]. Largely driven by usage-based approaches [7], a growing number of researchers have recently been concerned with the linguistic interpretation of specific syntactic structures for descriptive purposes [16–18], which is a prerequisite for a full understanding of linguistic features of learners’ written texts. Large-grained indices have difficulty in distinguishing a wide range of syntactic complexity features as they integrate multiple syntactic structures into a single set [7, 16, 18]. In this case, the interest in fine-grained indices that can distinguish structural subtypes of clauses and phrases is increasing.

Over the past two decades, academic writing has stood out in the field of L2 writing. Syntactic complexity in both published research articles and non-professional writing (e.g., coursework, thesis, and dissertation) has received extensive attention [19]. Numerous studies have claimed that academic writing is characterized by phrasal constructions and have reported a relative absence of clausal constructions [20–23]. Large-grained indices that have been adopted by the majority of scholars might not suffice in further investigation of the role of particular phrasal embedding and clausal elaboration in academic discourse. As a consequence, more studies that aim to investigate the syntactic complexity of academic writing through the use of fine-grained indices are needed, which can effectively complement analyses with large-grained indices [18]. On the other hand, it has been found that syntactic complexity in academic writing may be affected by various factors, such as the level of proficiency [12, 23, 24], topic [6, 25, 26], genre [5, 7, 11, 27], discipline [28–32], and first language (L1) background [33–39]. A few studies have recognized potential L1-related differences in the syntactic complexity of academic writing [33], commonly incorporating native speaker baselines to identify the gap in syntactic complexity between L1 and L2 groups. However, this study focuses on L1 and L2 writers with comparable proficiency levels. Moreover, extant studies have primarily accounted for one variable [33–36, 39], while multiple variables, such as language background and year, or language background and discipline, which can characterize more precise linguistic variation, remain underexplored. Collectively, this study serves the descriptive purpose to shed light on the differences in the clausal and phrasal complexity of L1 English and L1 Chinese students’ academic writing under the mediation of year and discipline through the use of fine-grained indices. The comparative approach adopted in this study will not only help determine how Chinese students deviate from (differ from) or approach (resemble) native students in a wide range of syntactic complexity features within academic writing but will also furnish valuable information for educators and institutions in the design of adequate instructional interventions tailored to academic writing programs based on existing characteristics [34].

## Syntactic complexity in academic writing

### Language background

A growing body of work has targeted both native and non-native speakers to scrutinize whether and how language background impacts syntactic complexity. The majority of these studies have adopted the same large-grained indices to assess the language output of L1 and L2 academic writing from the dimensions of length of production units, amount of subordination, amount of coordination, and degree of phrasal sophistication [33–37]. However, the results were not always consistent, which may be due to differences in their selection of research objects, writing tasks, and genres. In research articles, L2 experts appear to use longer production units and more complex nominals than L1 experts to achieve linguistic explicitness and conciseness [35]. In university-level academic writing, there is evidence that Chinese students tend to generate shorter utterances and employ fewer subordinate clauses and noun phrases than native students [34, 37]. Additionally, high-level L2 students approximate the syntactic patterns of L1 groups better than low-level L2 students [34]. However, some research did not explicitly observe statistically significant differences between L1 and L2 students in relation to syntactic complexity [33, 36], which may be partly attributed to treating L2 students with diverse language backgrounds as a unified group. This situation did not occur in the further measurements of Lu and Ai [33] when native speakers were compared separately with non-native speakers from seven different countries, highlighting the need to differentiate L2 writers with heterogeneous language backgrounds to prevent the blurring of language background effects.

Other research has opted for fine-grained indices primarily focusing on a variety of compressed constructions of noun phrases based on the assumption that a nominal style is more pronounced in formal academic writing [38, 39]. According to Lan et al. [39], L1 essays generally have diverse phrasal patterns, while more noun phrases are present in L2 essays. The construct of noun phrases in academic writing is also affected by language backgrounds, such as a higher frequency of pre-modifying nouns in L1 English texts and a greater proportion of prepositional phrases as postmodifiers in L1 Arabic texts [38]. Although these studies have extended previous findings, they both investigated only a particular discipline or disciplinary group [38, 39], potentially limiting the generalizability of the conclusions.

### Year

The development of syntactic complexity has been a central concern of L2 writing research. Numerous studies have followed the syntactic development trajectories of individuals or groups of learners over a period of time from a longitudinal perspective [8, 10, 11, 40–43], some of which have been conducted across years with a view to observing long-term changes in syntactic patterns [10, 42, 43]. There have also been cross-sectional studies that have investigated the syntactic features of learners at different grade levels, focusing on syntactic complexity differences across proficiency levels [2, 12–14, 23, 24].

Longitudinal and cross-sectional work on the development of syntactic complexity in academic writing has collectively revealed several trends. For instance, learners tend to pack meaning into phrasal constructions rather than clausal coordination and subordination as they accumulate academic writing experience and develop academic writing skills [11, 41]. In other words, scholarly texts produced by learners at the advanced level or in the mature stages of language development are more likely to feature noun phrases [10, 13, 42, 43]. Furthermore, the degree of phrasal sophistication increases with learning time and proficiency level [23, 24]. For example, Parkinson and Musgrave [24], who distinguished between the syntactic features of EAP students’ and MA students’ texts, found that the less proficient EAP group had a great reliance on attributive adjectives, which were hypothesized to be early-acquired phrasal modifiers. The more proficient MA group, in contrast, used more complex noun phrases modified by noun and prepositional phrases, resonating with the syntactic features of published research articles. In the present study, in addition to phrasal constructions, linguistic variation regarding specific clausal constructions was also captured.

### Discipline

Writing in academic contexts is governed by shared communicative purposes and endorsed communicative conventions among members of a given discourse community [44]. The use of community-sensitive linguistic resources is an important facet of effective scholarly communication as such research is more culturally appropriate to the community and thus more likely to persuade readers [28]. Consequently, reflecting on syntactic complexity in scholarly texts from a cross-disciplinary perspective has been favorably suggested. Prior research interest has centered on published research articles, particularly in the comparison of language conventions of research articles between soft and hard/pure and applied disciplines [28–32]. An emerging line of research has explored the relationship between syntactic complexity and rhetorical move-steps by analyzing the syntactic features used when achieving diverse goals of rhetorical functions [28, 29]. In lieu of a particular part-genre, Casal et al. [30] focused on RA part-genres (Introduction, Methods, Results, and Discussion), measuring syntactic complexity across disciplinary and part-genre variables.

Recently, some scholars have come to consider disciplinary variation when exploring students’ academic discourse [19, 27, 45]. For example, Dong et al. [19] mapped out disciplinary variation with respect to syntactic complexity in academic writing of L1 university students across 31 disciplines within 4 disciplinary families by means of large-grained indices, providing a relatively comprehensive and systematic picture of disciplinary features. Some parallel findings were identified in these studies. Generally, hard discipline texts contain a greater frequency of noun phrases, while soft discipline texts have a higher clausal complexity [27, 29, 30, 32]. To summarize, existing research taking a cross-disciplinary lens has mainly focused on the genre of research articles, with less attention paid to the specific syntactic structures and discourse functions of students’ written work. Subsequent research has been suggested to take discipline into account, given that it is a potential variable in shaping the profiling of academic language [46].

### Interaction of multiple factors

Current research that has addressed the effects of multiple factors on syntactic complexity in academic writing is relatively scarce [2, 10, 11, 27, 32, 45]. Ziaeian and Golparvar [32] paid attention to the interaction between language background and discipline by examining the syntactic structures in the discussion section of research articles published by L1 Persian and L1 English writers in the disciplines of Applied Linguistics, Economics, and Chemistry. The study claimed that L2 writers depended less on phrasal modifiers than L1 writers. However, this conclusion might be attenuated by the fact that L2 texts were mostly from novice writers, while L1 texts were collected from professional writers. Meanwhile, it pointed out that phrasal complexity occurred more frequently in Chemistry than in Applied Linguistics and Economics. When discussing the interplay of linguistic context and discipline, discipline can play a moderating role where L2 specialists from Applied Linguistics and Economics output a higher density and diversity of complex nominals. Staples et al. [45] responded to two questions regarding the developmental trajectory of linguistic complexity during the university years for L1 and L2 students and the mediating role of disciplines, annotating texts with Biber Tagger. In their study, the developmental trajectories of academic language for both native and non-native speakers shifted from divergence to convergence, using more phrases and fewer clauses in later stages. The study introduced a novel approach that relies on mean frequencies and CIs to illustrate the trajectory of linguistic development, bringing fresh insights to the domain.

In contrast to the study by Staples et al. [45], the present study places emphasis on how multiple factors, including language background, year, and discipline, interact with each other in academic writing, rather than treating the trajectory of linguistic development as a baseline. Moreover, they approached L2 students from diverse countries as a singular entity; however, syntactic complexity is sensitive to participants’ linguistic contexts [33, 39]. Hence, this study restricts the educational context of L2 learners to one nation, China, in an effort to offer more inspirations that build upon prior research. Upon conducting an exhaustive review of existing literature, it becomes evident that these investigations have made substantial contributions to syntactic complexity in academic writing. Nevertheless, there remains ample room for further exploration. Firstly, the objective of this study is to elucidate the syntactic forms and functions within academic writing through the identification of fine-grained clausal and phrasal indices, an aspect which has often lacked adequate attention. Additionally, research into the influences of multiple factors on syntactic complexity in academic writing is relatively limited and insufficient. Therefore, this study refines L1 and L2 groups through years and disciplines to compare the syntactic complexity of academic writing across university years and diverse disciplines for both English native speakers and Chinese English learners. The research questions are as follows.

1. Are there differences in the syntactic complexity of English academic writing produced by L1 English and L1 Chinese students as the year progresses? If so, what are the differences?
2. Are there differences in the syntactic complexity of English academic writing produced by L1 English and L1 Chinese students within or among disciplines? If so, what are the differences?
3. Is there a significant interaction between language background, year, and discipline? If so, what are the specific features?

## Methods

### Corpus

All the data collected and analyzed in this study were drawn from the British Academic Written English Corpus (BAWE), which contains academic writing from students at different UK universities. The corpus covers good-standard (Distinction and Merit) academic texts of various genres (e.g., essay, critique, methodology recount, and case study) written by students from L1 English and multiple L1 non-English countries throughout four years of study (first-year, second-year, third-year university, and taught masters) and across four discipline groups (Life Sciences, Physical Sciences, Social Sciences, and Arts and Humanities).

Based on the research focus, this study only examined written texts from L1 English and L1 Chinese speakers. Due to the lack of Chinese student samples in the Arts and Humanities, we focused on the Life Sciences (LS), Physical Sciences (PS), and Social Sciences (SS). Also, because the number of texts from L1 English writers far exceeded that of L1 Chinese writers in the corpus, to avoid the detrimental effects of uneven sample size, we used SPSS to randomly sample the L1 pool to ensure that the number of L1 texts in each year and discipline matched that of the L2 texts. Ultimately, a total of 472 academic writings made up this study corpus, including two sub-corpora from L1 and L2. The L1 sub-corpus contains randomized 236 writing texts generated by native speakers from four years of LS, PS, and SS disciplines, and the L2 sub-corpus comes from the same amount of academic writing by Chinese students. The number of texts, total word count, and mean word count for each year and discipline of the two groups are presented in **Table 1**. Other considerations regarding sub-corpora are also mentioned. This corpus is evidently incapable of providing a platform for a longitudinal study, and it is considered to be a cross-section from the undergraduate to graduate levels. Additionally, the study did not narrow down the genres of the two sub-corpora, mainly on account of the need for sufficient sample size and the different genre preferences among disciplines, e.g., more case studies in the Life Sciences, methodology recount in the Physical Sciences, and essays in the Social Sciences. Lastly, given the characteristics of BAWE that all texts gathered were rated to a good standard, it is necessary to reiterate that the aim of the study is not to identify the gap between syntactic complexity in Chinese-L1 students’ academic writing and that of English-L1 students but to interpret to what extent syntactic features are influenced by linguistic backgrounds and whether this influence is mediated by year and discipline.

**Table 1:** Description of the corpus in this study.

		L1 Group			L2 Group		
		Number of text	Total words	Mean words	Number of text	Total words	Mean words
**Y1**	**LS**	21	37,176	1770	21	23,410	1115
	**PS**	12	18,158	1513	12	17,566	1464
	**SS**	17	31,841	1873	17	31,805	1871
	*Subtotal*	50	87,175	1719	50	72,781	1483
**Y2**	**LS**	17	33,829	1990	17	28,654	1686
	**PS**	13	29,242	2249	13	24,124	1856
	**SS**	10	27,222	2722	10	25,011	2501
	*Subtotal*	40	90,293	2320	40	77,789	2014
**Y3**	**LS**	20	46,597	2330	20	41,706	2085
	**PS**	20	47,714	2386	20	57,513	2876
	**SS**	22	63,934	2906	22	56,986	2590
	*Subtotal*	62	158,245	2541	62	156,205	2517
**Y4**	**LS**	15	39,303	2620	15	26,745	1783
	**PS**	25	50,344	2014	25	87,772	3511
	**SS**	44	153,941	3615	44	148,166	4609
	*Subtotal*	84	243,588	2750	84	262,683	3301
	*Total*	236	579,301	2333	236	569,458	2329

### Fine-grained indices of syntactic complexity

The Tool for the Automatic Analysis of Syntactic Sophistication and Complexity (TAASSC) developed by Kyle [47] served as a proxy in this study for automated annotation of linguistic features of large-scale texts. This tool measures not only large-grained syntactic complexity but also fine-grained causal and phrasal complexity. TAASSC was chosen rather than other fine-grained analyzers (e.g., Biber Tagger or Coh-Metrix) because of its wide availability and batch processing capabilities, covering 31 causal complexity indices and 132 phrasal complexity indices, without treating clauses and phrases as two integrated variables. Furthermore, the tool uses state-of-the-art parsers with an accuracy of around 90% [16]. For instance, it calculates clause and phrase length as the number of direct dependents per clause/nominal, rather than the word count, thereby preventing longer sentences from receiving undue weight.

#### Indices of clausal complexity

We did not employ all the indices provided by TAASSC due to the potential inflation of Type-I errors that could arise from testing a multitude of indices [48]. Instead, we judiciously selected the optimal indices for this study based on multidimensional linguistic constructs to enable in-depth observation and analysis. This study established a framework based on Biber et al.’s [18] proposed taxonomy of structural types and sentential functions, encompassing major features of English grammar. The structural types can be categorized into three classes: finite dependent clauses, non-finite dependent clauses, and dependent phrases, all of which can be captured through indices within TAASSC (see **Table 2**). Among these types, finite and non-finite dependent clauses exhibit three syntactic functions: adverbial, complement, and noun modifier [18]. In this study, finite and non-finite adverbial clauses, such as conditional, causal, concessive, and purposive adverbial clauses, can be measured using the adverbial clause index. Finite complement clauses, including that-clauses controlled by verbs, nouns, and adjectives, and wh-clauses controlled by verbs, are identified by the clausal complement index. Non-finite complement clauses accompanied by to-clauses controlled by verbs, nouns, and adjectives, as well as ing-clauses controlled by verbs, can be captured by the open clausal complement. Finite noun modifier clauses that refer to relative clauses with that- or wh- can be interpreted as relative clause modifiers in TAASSC; however, this category has been classified by Kyle [47] under phrasal complexity indices as relative clauses function as modifiers of head nouns. In addition to these, this study also incorporated two overarching indices, namely, dependents per clause (cl_av_deps) and the standard deviation of dependents per clause (cl_ndeps_std_dev), to respectively detect overall clausal sophistication and diversity.

**Table 2:** Clausal and phrasal dependent types measured in this study.

Syntactic structure	Abbreviation	Example
***Clausal complexity***		
adverbial clause	advcl	She felt much better **when she took a long nap in the afternoon**.
clausal complement	ccomp	She believed **that she had made the right decision to accept the job offer**.
open clausal complement	xcomp	I am eager **to learn new skills**.
		She saw him **standing on the corner**.
***Phrasal complexity***		
adjectival modifiers	amod	The **old** house was in need of repairs.
prepositional phrases	prep	The book **on the shelf** caught her eye.
verbal modifiers	vmod	The excited dog **wagged** its tail vigorously.
nouns as modifiers	nn	The **soccer** ball rolled across the field.
relative clause modifiers	rcmod	The woman **who lives next door** is a doctor.
adverbial modifiers	advmod	The **extremely** tall man ducked his head.

#### Indices of phrasal complexity

TAASSC provides a sufficient array of phrasal complexity indices, which can be broadly categorized into three groups: phrase types (e.g., nominal subjects), particular dependent types (e.g., prepositional phrases), and particular dependent types associated with phrase types (e.g., prepositional phrases in nominal subjects). Given the special focus of this study on hypothesized complex nominals as a marker of advanced academic writing, only the second category of phrasal complexity indices was utilized, comprising different parts of speech (POS) modifying nominal phrases. These involve attributive adjectives, post-modifying prepositional phrases, non-finite verbs or verb phrases, pre-modifying nouns, non-clausal adverbs, and that- or wh-relative clauses (see **Table 2**). Alongside these six dependent types, two general phrasal complexity indices were similarly identified, namely, dependents per nominal (av_nominal_deps) and the standard deviation of dependents per nominal (nominal_deps_stdev), corresponding to phrasal sophistication and diversity.

### Data analysis

The study measured the interaction effects of language background and year, language background and discipline, as well as language background, year and discipline on clausal and phrasal complexity through two-factor and three-factor ANOVA operationalizations, respectively. Prior to manipulation, Shapiro-Wilk for normality and Levene’s test for equality of variances were conducted. Out of 8 indices met the assumptions of the ANOVA, and the remaining 5 indices (vmod; rcmod; advmod; advcl; ccomp) did not. After the sqrt transformation of the five indices, all data showed normal distribution and homogeneity of variance. The indices that yielded significant differences in the analysis were further conducted with univariate tests and pairwise comparisons adjusted by Bonferroni to observe specific variations. Effect sizes were based on Cohen [49], with thresholds of 0.01, 0.06, and 0.14 for small, medium, and large effects of η^2^.

## Results

### Comparison of L1 English and L1 Chinese students across years

Factorial MANOVA revealed that background (Wilk’s Λ = .700; F(13, 452) = 14.923, *p* < 0.001, η^2^ = .300) and year (Wilk’s Λ = .748; F(39, 1339) = 3.535, *p* < 0.001, η^2^ = .092) had a significant main effect on the syntactic complexity and that there was an interaction effect (Wilk’s Λ = .841; F(39, 1339) = 2.071, *p* < 0.001, η^2^ = .056). Therefore, follow-up univariate analyses were performed to trace the impact on each index, as presented in **Table 3**. The results showed an interaction between background and year for 6 of the 13 indices, of which 3 indices (cl_av_deps, cl_ndeps_std_dev, and av_nominal_deps) represent the global complexity and 3 indices (nn, rcmod, and advmod) are nominal phrases. The study further examined simple effects of background baseline within each level in combination with other effects and pairwise comparisons using Bonferroni adjustment to determine between-group differences for each year.

**Table 3:** Results of a two-factor ANOVA: Background * year.

Dependent variable	Source	*df*	*F*	*p*	η^2^
cl_av_deps	background	1	.211	.646	.000
	year	3	3.731	.011*	.024
	background * year	3	4.825	.003**	.030
cl_ndeps_std_dev	background	1	.191	.662	.000
	year	3	7.360	< .001***	.045
	background * year	3	4.275	.005**	.027
advcl	background	1	17.433	< .001***	.036
	year	3	5.562	.001**	.035
	background * year	3	2.017	.111	.013
ccomp	background	1	14.360	< .001***	.030
	year	3	.678	.566	.004
	background * year	3	1.816	.143	.012
xcomp	background	1	16.953	< .001***	.035
	year	3	.358	.783	.002
	background * year	3	.829	.479	.005
av_nominal_deps	background	1	2.722	.100	.006
	year	3	9.018	< .001***	.055
	background * year	3	4.236	.006**	.027
nominal_deps_stdev	background	1	1.045	.307	.002
	year	3	4.133	.007**	.026
	background * year	3	.398	.754	.003
amod	background	1	4.359	.037*	.009
	year	3	7.475	< .001***	.046
	background * year	3	1.641	.179	.010
prep	background	1	.202	.653	.000
	year	3	4.268	.005**	.027
	background * year	3	1.239	.295	.008
vmod	background	1	38.987	< .001***	.078
	year	3	.118	.950	.001
	background * year	3	.412	.745	.003
nn	background	1	28.852	< .001***	.059
	year	3	8.934	< .001***	.055
	background * year	3	3.993	.008**	.025
rcmod	background	1	63.094	< .001***	.120
	year	3	4.717	.003**	.030
	background * year	3	4.785	.003**	.030
advmod	background	1	16.996	< .001***	.035
	year	3	2.626	.050	.017
	background * year	3	3.205	.023*	.020

Note

*p < 0.05

**p < 0.01

***p < 0.001

**Fig 1:**
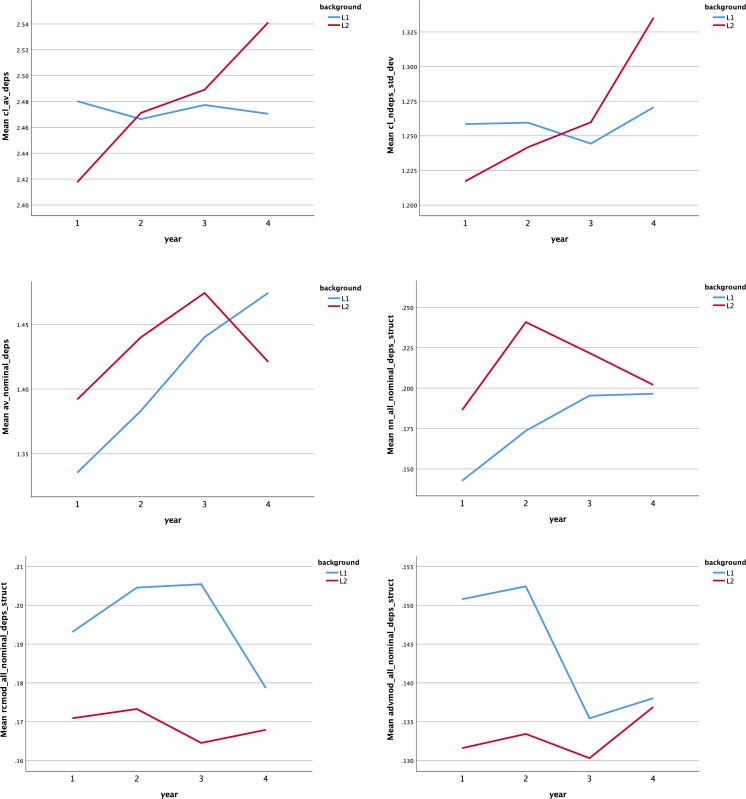
Means of six indices that interacted between background and year. The six indices are: (a) dependents per clause; (b) dependents per clause (standard deviation); (c) dependents per nominals; (d) nouns as a nominal dependent per nominal; (e) relative clause modifiers per nominal; (f) adverbial modifiers per nominal.

**Fig 1** shows the means of six indices that interacted between background and year. For dependents per clause, significant differences were found in the 1st (*F* = 5.018, η^2^ = .011) and 4th (*F* = 10.716, η^2^ = .023) year, with more use by L1 English students than L1 Chinese students in the first year (*p* < 0.05), and vice versa in the fourth year (*p* < 0.01). When it comes to clausal diversity, also known as dependents per clause (standard deviation), texts from L1 Chinese writers were more varied in clauses in the final year than L1 English writers’ texts (*F* = 11.036, *p* < 0.01, η^2^ = .023). Similarly, the statistical change occurred only in the fourth year, but the difference was that dependents per nominals were written more by L1 English students than by L1 Chinese students (*F* = 5.333, *p* < 0.05, η^2^ = .011). It is evident that L1 Chinese writers produced more noun modifiers than L1 English writers until the third year (*F* = 9.872, *p* < 0.01, η^2^ = .021; *F* = 18.671, *p* < 0.001, η^2^ = .039; *F* = 4.454, *p* < 0.05, η^2^ = .010). The feature of relative clause modifiers in L1 writing was more prominent than those in L2 writing throughout the four years of study (*F* = 10.290, *p* < 0.01, η^2^ = .022; *F* = 16.287, *p* < 0.001, η^2^ = .034; *F* = 43.153, *p* < 0.001, η^2^ = .085; *F* = 4.088, *p* < 0.05, η^2^ = .009). There were also significant differences between the two groups in terms of adverbial modifiers, with higher frequency in L1 texts in the 1st and 2nd year (*F* = 11.559, *p* < 0.01, η^2^ = .024; *F* = 9.072, *p* < 0.01, η^2^ = .019).

### Comparison of L1 English and L1 Chinese students within and among disciplines

According to multivariate tests, there was a main effect of background (Wilk’s Λ = .703; F(13, 454) = 14.743, *p* < 0.001, η^2^ = .297) and discipline (Wilk’s Λ = .588; F(26, 908) = 10.604, *p* < 0.001, η^2^ = .233) on clausal and phrasal complexity, and an interaction effect (Wilk’s Λ = .870; F(26, 908) = 2.515, *p* < 0.001, η^2^ = .067) was also found. As illustrated in **Table 4**, the interaction effects of background and discipline appeared significantly with clausal indices of dependents per clause and clausal complements, as well as phrasal indices of dependents per nominal, prepositional modifiers, and relative clause modifiers.

**Table 4:** Results of a two-factor ANOVA: Background * discipline.

Dependent variable	Source	*df*	*F*	*p*	η^2^
cl_av_deps	background	1	1.081	.299	.002
	discipline	2	42.469	< .001***	.154
	background * discipline	2	5.281	.005**	.022
cl_ndeps_std_dev	background	1	1.723	.190	.004
	discipline	2	19.502	< .001***	.077
	background * discipline	2	.967	.381	.004
advcl	background	1	14.609	< .001***	.030
	discipline	2	.486	.615	.002
	background * discipline	2	.303	.739	.001
ccomp	background	1	14.804	< .001***	.031
	discipline	2	31.740	< .001***	.120
	background * discipline	2	6.139	.002**	.026
xcomp	background	1	16.256	< .001***	.034
	discipline	2	2.408	.091	.010
	background * discipline	2	2.873	.058	.012
av_nominal_deps	background	1	.484	.487	.001
	discipline	2	22.729	< .001***	.089
	background * discipline	2	4.920	.008**	.021
nominal_deps_stdev	background	1	1.218	.270	.003
	discipline	2	4.452	.012*	.019
	background * discipline	2	2.600	.075	.011
amod	background	1	2.478	.116	.005
	discipline	2	.439	.645	.002
	background * discipline	2	.838	.433	.004
prep	background	1	.665	.415	.001
	discipline	2	7.157	.001**	.030
	background * discipline	2	7.625	.001**	.032
vmod	background	1	40.474	< .001***	.080
	discipline	2	3.837	.022*	.016
	background * discipline	2	1.063	.346	.005
nn	background	1	19.756	< .001***	.041
	discipline	2	24.604	< .001***	.096
	background * discipline	2	1.996	.137	.008
rcmod	background	1	56.612	< .001***	.108
	discipline	2	1.085	.339	.005
	background * discipline	2	6.879	.001**	.029
advmod	background	1	12.037	.001**	.025
	discipline	2	11.250	< .001***	.046
	background * discipline	2	1.295	.275	.006

**Fig 2:**
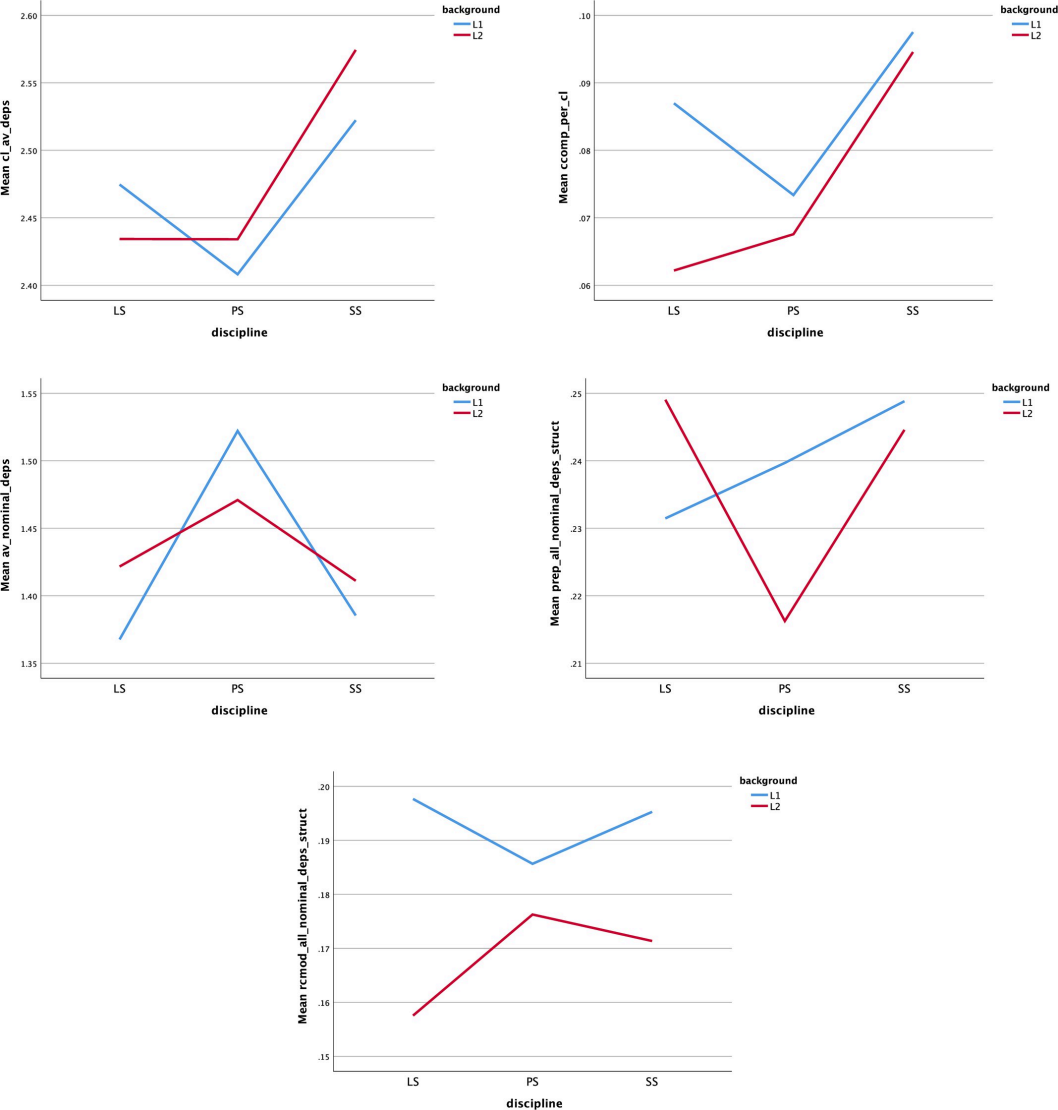
Means of five indices that interacted between background and discipline. The five indices are: (a) dependents per clause; (b) clausal complements per clause; (c) dependents per nominals; (d) prepositions per nominal; (e) relative clause modifiers per nominal.

**Fig 2** illustrates the means of five indices that interacted between language background and discipline. Pairwise comparisons showed significant differences in dependents per clause in SS, where Chinese-L1 writers used more clauses than English-L1 writers (*F* = 7.421, *p* < 0.01, η^2^ = .016). This index was higher in SS than in other disciplines for both backgrounds. Between-group differences revealed that L1 English students tended to produce more clausal complements than L1 Chinese students in academic texts in LS (*F* = 24.047, *p* < 0.001, η^2^ = .049), while within-group differences reflected high outputs of this measure for both L1 and L2 backgrounds in the SS discipline. The L2 group showed a higher frequency of producing dependents per nominal in LS (*F* = 4.920, *p* < 0.05, η^2^ = .010), while the L1 group tended to use it more in PS (*F* = 4.241, *p* < 0.05, η^2^ = .009). Both groups had higher phrasal complexity in the PS discipline. Significant differences between L1 English and L1 Chinese students’ prepositional modifiers index were found in LS and PS, with a lower frequency appearing in L1 academic texts of LS (*F* = 5.710, *p* < 0.05, η^2^ = .012) and vice versa in PS (*F* = 9.722, *p* < 0.01, η^2^ = .020). In addition, L1 writing exhibited a higher frequency of relative clause modifiers than L2 writing in both LS (*F* = 47.794, *p* < 0.001, η^2^ = .093) and SS (*F* = 21.639, *p* < 0.001, η^2^ = .044).

### Interaction between language background, year, and discipline

**Fig 3:**
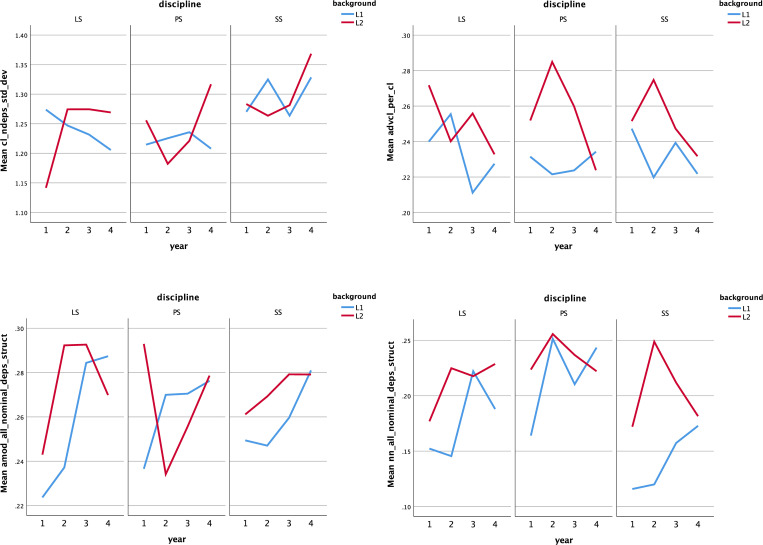
Means of four indices that interacted between background, year, and discipline. The four indices are: (a) dependents per clause (standard deviation); (b) adverbial clauses per clause; (c) adjectival modifiers per nominal; (d) nouns as a nominal dependent per nominal.

A three-factor ANOVA (see **Table 5**) revealed an interaction effect between language background, year, and discipline (Wilk’s Λ = .720; F(78, 2410) = 1.893, *p* < 0.001, η^2^ = .053) and further explored four indices, including dependents per clause (standard deviation), adverbial clauses, adjectival modifiers, and noun modifiers. **Fig 3** manifests the variation in the mean values of the four significant indices over four years and across three disciplines. Pairwise comparisons demonstrated the differences between students from the two backgrounds across different disciplines and years in relation to clausal diversity. Specifically, the statistics for native speakers were significantly higher than those for students from China in the LS discipline during the first year (F = 12.580, *p* < 0.001, η^2^ = .027), but no difference was observed in the next three years. In PS, L2 texts showed more diverse clause structures than L1 texts only in the fourth year (F = 10.146, *p* < 0.01, η^2^ = .022). There was no difference between students from both backgrounds in SS over four years.

**Table 5:** Results of a three-factor ANOVA: Background * year * discipline.

Dependent variable	Background	Year	Discipline	Background * Year	Background * Discipline	Year * Discipline	Background * Year * Discipline
cl_av_deps	.800	.146	< .001***	.004**	.060	.271	.294
cl_ndeps_std_dev	.472	.039*	< .001***	.007**	.700	.120	.019*
advcl	< .001***	.005**	.998	.059	.655	.864	.011*
ccomp	< .001***	.608	< .001***	< .001***	.007**	.009**	.657
xcomp	< .001***	.866	.182	.701	.050	.023*	.555
av_nominal_deps	.076	< .001***	< .001***	.011*	.013*	.323	.140
nominal_deps_stdev	.343	.060	.024*	.796	.091	.010*	.352
amod	.056	.001**	.956	.113	.542	.089	.028*
prep	.371	.039*	.010*	.540	.001**	.012*	.270
vmod	< .001***	.879	.152	.717	.308	.006**	.973
nn	< .001***	< .001***	< .001***	.006**	.018*	.185	.024*
rcmod	< .001***	.001**	.128	< .001***	.003**	.019*	.136
advmod	< .001***	.011*	< .001***	.018*	.618	.043*	.073

Exploring the index of adverbial clauses, there were significant differences in the first and third years of LS (F = 4.302, *p* < 0.05, η^2^ = .010; F = 8.093, *p* < 0.01, η^2^ = .018), the second and third years of PS (F = 10.598, *p* < 0.01, η^2^ = .023; F = 5.286, *p* < 0.05, η^2^ = .012), and the second year of SS (F = 6.122, *p* < 0.05, η^2^ = .013), all of which had more production from Chinese-L1 than English-L1 writers. The difference regarding adjectival modifiers existed only for the second-year LS discipline (F = 8.542, *p* < 0.01, η^2^ = .019) and first-year PS discipline (F = 6.296, *p* < 0.05, η^2^ = .014), again which showed a higher index for Chinese writers. The variation in the feature of nouns as modifiers were mainly in the 2nd year of LS (F = 12.580, *p* < 0.001, η^2^ = .027), the 1st year of PS (F = 5.013, *p* < 0.05, η^2^ = .011), and the first three years of SS (F = 6.324, *p* < 0.05, η^2^ = .014; F = 19.532, *p* < 0.001, η^2^ = .042; F = 7.690, *p* < 0.01, η^2^ = .017), and all were higher for L2 English learners.

## Discussion

In light of the substantial findings, the present study reveals the prevalence of syntactic complexity features in the academic writing of L1 English and L1 Chinese students across different years and disciplines, expanding and echoing the picture depicted by previous research. To answer the first research question, measures of the majority of clausal and phrasal structures in academic texts of L1 English and L1 Chinese students across years did not reveal significant differences, except for three phrasal indices of relative clause modifiers, nouns as modifiers, and adverbial modifiers. Relative clause modifiers and adverbial modifiers were more prevalent in native speakers’ written texts, in line with Ziaeian and Golparvar’s [32] finding that L1 experts used these constructions to a greater extent in research articles than L2 counterparts. Nouns as modifiers were more commonly seen in Chinese students’ written texts, especially in the early stages. Some researchers based on qualitative analyses have claimed that L2 learners rely more on lexico-grammatical chunks, particularly chunks based on pre-modifying nouns [27, 39]. However, their early production of pre-modifying nouns was dominated by repeated forms, and the more advanced or experienced writers tended to use fewer repeated lexico-grammatical chunks [24, 39]. In the fourth year of university, only the index of relative clause modifiers differed significantly in the academic writing of L1 English and L1 Chinese students, which somewhat supports the conclusion of Staple et al. [45] that the grammatical trajectories of L1 and L2 writers moved from divergence towards convergence.

In terms of the global complexity of clauses and phrases, academic written texts from the L1 and L2 groups did not show significant differences in the early years. However, in the final year of university, L1 English students’ writing was characterized by more noun phrases, and L1 Chinese students’ writing was embodied in more diverse and denser clausal structures. The discovery of variation in global complexity revealed that L1 English students tended to use phrasal structures to achieve a high density of information in academic writing rather than clausal embedding as proficiency increased in comparison to L1 Chinese students. Additionally, within-group comparisons enhance the persuasiveness of this finding. The comparisons demonstrated a consistent rise in the frequency of complex nominal occurrences within texts written by L1 English students, juxtaposed with a decline in texts from L1 Chinese students. However, it is important to note that there still remained a noteworthy increase for L1 Chinese students in the fourth year compared to the first year. The greater reliance on noun phrases as the year progresses can be interpreted as an indication of students’ mounting need for precise language, especially when confronted with more specialized disciplinary knowledge.

In response to the second inquiry, it was observed that native English speakers displayed a tendency to produce a greater number of complement clauses and relative clause modifiers, while native Chinese students tended to favor the use of more post-modifying prepositional phrases in the field of Life Sciences. However, when it comes to the realm of Physical Sciences, L1 academic texts demonstrated a notably higher frequency of post-modifying prepositional phrases compared to their L2 counterparts. In the domain of Social Sciences, Chinese English learners relied more heavily on clausal structures, whereas native speakers consistently employed relative clauses to extensively modify nouns. Interestingly, despite the varying usage preferences between L1 English and L1 Chinese students within the same discipline, both groups simultaneously showcased comparable syntactic features across disciplines. For instance, clausal structures, especially complement clauses, were prominently manifested in the Social Sciences, whereas noun phrases prevailed in the Physical Sciences. The results align with the disciplinary variation in syntactic complexity identified by many studies [27, 29, 30, 32] and are likely attributed to the distinct discourse demands of the respective disciplines. Students in the Physical Sciences necessitate succinctly portraying experimental procedures and interpreting results with mathematical formulations [32]. This efficiency is facilitated through the use of compressed noun phrases, which imbue clarity and objectivity into their expressions. On the other hand, the Soft Sciences favor nuanced language for articulating arguments and constructing positions, which leads to the frequent use of complex subordinate clauses to enhance content coherence and logical presentation. Within the context of Social Sciences, the prevalence of clausal complements significantly outstripped other disciplines. Complement clauses serve to relay one’s stance or that of others, elaborating information as supporting evidence and affording writers the means to convey arguments [50]. The grammatical developmental trajectory proposed by Biber and Gray [21] posits the usage of subordinate clauses as an initial or early stage of academic writing. However, this study indicates that the efficacy of academic language cannot be merely encapsulated within noun phrases, underscoring the significance of accounting for the disciplinary attributes when evaluating the syntactic complexity of academic writing.

Turning attention to the third query, it became evident that there was no significant discrepancy in the global complexity of clauses and phrases within academic writing texts written by L1 English and L1 Chinese writers. However, certain aspects of Chinese English learners’ syntactic development warrant specific attention. Adverbial clauses were found to characterize the syntactic features of Chinese students, echoing prior research [51, 52]. Furthermore, in comparison to native English students, native Chinese students exhibited a higher frequency of using adjective modifiers and nouns as modifiers in early Life and Physical Sciences texts, as well as nouns as modifiers in three-year undergraduate essays in the Social Sciences. In phrasal structures, Chinese English learners prefer to use attributive adjectives and pre-modifying nouns to modify nouns, which is in agreement with Cao and Xiao [53]. In Mandarin Chinese, a basic principle of word order is the modifying-modified sequence, such as possessive, adjective, and relative clauses preceding nouns [51, 54]. Also, nouns can only be pre-modified in Chinese [54]. Therefore, native language transfer may contribute to Chinese students maintaining their preference for L1 modifiers in L2 academic writing. Drawing from Biber and Gray’s [21] framework, nouns and adjectives as modifiers of the head noun are exceedingly common in written academic registers. These findings seem to provide evidence for the idea that L2 writers tend to adapt to academic language conventions earlier than L1 writers [45]. This study re-emphasizes the need to examine syntactic complexity under a multifactorial context.

## Conclusion

This study enriches the line of research on syntactic complexity in L1 or L2 academic writing by incorporating native language cohorts into a comparative analysis. It elucidates both similarities and differences in the linguistic features of English academic writing between L1 English and L1 Chinese students. The developmental trajectories of syntactic complexity may differ among students from distinct L1 backgrounds, even when operating at comparable proficiency levels [33]. The written texts of native English-speaking students were closer to the syntactic features of advanced academic writing compared to native Chinese-speaking students as the years progressed. Overall, however, the use of noun phrases gradually increased for both groups of students. The study suggests the establishment of a temporal baseline to track the syntactic complexity of academic writing because syntactic complexity development tends to be nonlinear, intricate, and dynamic [55]. Additionally, the study highlights similar syntactic characteristics across disciplines among students from two different L1 backgrounds. The common tendency involves a greater reliance on compressed noun phrases within the Physical Sciences and a frequent utilization of complex clauses within the Social Sciences. Lastly, the study reveals an interaction between language background, year, and discipline. Within this interplay, Chinese English learners manifest certain early markers of academic language, including adjective modifiers and nouns as modifiers.

Comparing the syntactic features of L1 English and L1 Chinese students holds meaningful implications for EAP instruction. It equips educators and learners with a more comprehensive, precise, and profound grasp of the linguistic conventions within academic writing. In the pursuit of enhancing students’ conscious incorporation of academic language, particularly noun phrases, instructors ought to begin by imparting students exhaustively regarding pertinent grammatical systems, such as dependent types (e.g., attributive adjectives, pre-modifying nouns, post-modifying prepositional phrases, and relative clauses), alongside phrase types (e.g., nominal subjects or direct objects). Furthermore, students are encouraged to revise their own academic work under the guidance of teachers, with a specific focus on how loosely structured content can be reconfigured into denser and information-centric forms anchored by noun phrases. This practical exercise aids in deepening their comprehension of academic writing features. Simultaneously, educators should not overlook the language discrepancies inherent in different academic disciplines. They should orchestrate a range of discipline-specific instructional activities to elucidate the distinct functions and communicative purposes conveyed by clauses and phrases, such as explanatory power and explicitness, to cater to the diverse disciplinary features of academic writing. Additionally, educators who teach Chinese students should recognize the syntactic traits exhibited in the academic writing of native speakers, along with the similarities and differences in performance observed among Chinese English learners. Interventions should be tailored in alignment with the syntactic preferences of Chinese students, which have been shaped by cultural, linguistic, and academic backgrounds, rather than imposing native norms into the L2 context. For instance, an augmentation of students’ awareness and practice concerning particular noun phrase structures, including adjective modifiers and noun modifiers, could potentially expedite their adaptation to linguistic features as they initiate their engagement with academic writing.

The limitations of the study also need to be mentioned. First, the sample sizes collected by the study at each level may not be large enough, which probably limits the generalizability of the results to a larger target group. This is mainly determined by the number of Chinese students’ texts in the corpus. Furthermore, the study selected major indices from TAASSC, which may not cover all common syntactic structures in academic writing. Therefore, a larger sample size and more comprehensive indices will be included in future studies in order to further compare the academic syntactic complexity of students from diverse L2 countries and L1 backgrounds.
